# Issues of accessibility to health services by older Australians: a review

**DOI:** 10.1186/s40985-018-0097-4

**Published:** 2018-07-16

**Authors:** Deborah van Gaans, Elsa Dent

**Affiliations:** 1 Centre for Population Health Research, School of Health Sciences, University of South Australia, GPO Box 2471, Adelaide, South Australia Australia; 2 Centre for Positive Ageing and Wellbeing, Torrens University Australia, Adelaide, South Australia Australia; 3 Baker Heart and Diabetes Research Institute, Melbourne, Victoria Australia

**Keywords:** Accessibility, Aged, Older people, Health services, Public health, Equity

## Abstract

**Background:**

This review provides an in-depth investigation into the difficulties facing older Australians when accessing health care services.

**Methods:**

A literature search was conducted in December 2016 using Academic Premier to identify relevant publications. Key search terms were accessibility, health service, older people and Australia. Papers published between 1999 and 2016 were included. Statements of accessibility were extracted and then grouped using the five dimensions of accessibility by Penchansky and Thomas (1981): availability, accessibility, accommodation, affordability and acceptability.

**Results:**

Forty-one papers were included. Availability issues identified were inadequate health care services, particularly for culturally and linguistically diverse (CALD) populations and those residing in rural areas. Accessibility issues included difficulties accessing transport to health care services, which in turn restricted choice of appointment time. Issues of accommodation identified were long waiting times for appointments with both general practitioners and medical specialists. Affordability was a common problem, compounded by multi-morbidity requiring high health care use. Issues of acceptability centred on the role of the family, feelings of shame when receiving care from a non-family member, traditional practices and gender sensitivity.

**Conclusions:**

The contribution of factors to health service accessibility varies according to an older person’s geographical local and their accessibility to transport, as well as their level of multi-morbidity and cultural background. Improving access to health services could be improved by matching services to the population that they serve.

## Background

Over the past century, average Australian mortality rates have fallen significantly, with life expectancies rising for both men and women [1]. This fall in mortality rate has added to population growth and the proportion of older people in the Australian population. In 2015, there were an estimated 3.5 million older Australians, representing one in every seven people (15.1%); this proportion has increased from 13.3% in 2009 to 14.3% in 2012 [2].

The impending rapid growth of Australia’s older population has important implications for provision of services needed by older people [3]. Our ageing population challenges the ability of health services to maintain health and wellbeing, manage serious and continuing illness and provide support for older people with frailty and/or disability [4]. This challenge is not only because there are many more Australians surviving to old age than was the case for previous generations, but it may well be that on average they have more co-morbidities requiring higher levels of care than earlier generations [3].

In 2015, the Australian Bureau of Statistics identified that 94.8% of older people lived in households [2]. This highlights Australia’s federal government policy of ‘ageing in place’, wherein the objective is to maintain older people in the community for as long as possible, whilst reducing morbidity, hospitalisation and admission to aged care services [5]. Ageing in place aims to provide support for older people so that they can live where they choose for as long as they can. As the population of Australia ages, it is critical that older adults, their families, healthcare providers and healthcare systems are equipped to deal with the challenges of cultural diversity and heterogeneity in ageing. Concomitantly, arrangements for the delivery of Australian aged care services have changed dramatically in recent times [6].

As the length of life and proportion of older persons increase in most industrialised and many developing nations, a central question is whether this population ageing will be accompanied by sustained or improved health, improved quality of life and sufficient social and economic resources [7]. Access to appropriate and adequate services has the potential to minimise risk and enhance well-being and, in turn, reduce the need for acute hospital admissions and delay entry into long-term residential care [8]. A range of factors interact to influence a patient’s ability to access health care at any point in time. Penchansky and Thomas have defined the following five dimensions to describe accessibility: availability, accessibility, accommodation, affordability and acceptability [9].

## Method

### Search strategy

The review was undertaken in December 2016 and used the *Academic Search Premier* database to identify relevant articles. This multidisciplinary research database provides access to academic journal articles, newspapers and magazines. Key search terms were purposely kept broad and included ‘accessibility’, ‘health service’, ‘older people’ and ‘Australia’.

### Inclusion and exclusion criteria

Only English-language papers published between 1999 and 2016 were included. Abstracts were screened by the first author and discarded if the article did not contain any information on the accessibility of older Australians to healthcare services. The full texts for all selected abstracts were retrieved and then assessed for inclusion by both authors. Any disagreement over inclusion of the full articles was resolved through face-to-face meetings. A lateral search was also performed by both authors to identify additional articles using the reference lists of identified articles. No further studies were identified during the lateral search.

Inclusion criteria included publication in peer-reviewed academic journals. Because we were looking for specific *issues* related to healthcare accessibility for older Australians, no restriction was placed upon what publication type. Accordingly, our review included original articles, systematic reviews, opinion pieces and editorials published in academic journals. Regarding participants, we included all publications involving and/or discussing older Australians (aged 65 years and over). Studies including migrants and Indigenous older Australians were also included, with an age ≥ 50 years defining ‘older person’ for Indigenous Australians. Excluded were papers focusing outside of Australia and studies of health service accessibility not specifically including older adults.

### Data extraction and analysis

Both authors agreed upon the search strategy and the inclusion/exclusion criteria before the study’s inception. Full papers identified during the literature search were exported by the first author into an Excel spreadsheet for analysis. The included papers were sorted by both authors into Penchansky and Thomas’ five dimensions of accessibility: availability, accessibility, accommodation, affordability and acceptability [9], as defined below:

- *Availability* as the relationship between the volume and type of existing services (and resources) and the clients’ volume and types of needs. Availability refers to the adequacy of the supply of physicians, dentists and other providers or facilities, such as clinics and hospitals and of specialised programs and services, such as mental health and emergency care [9];
- *Accessibility* as the relationship between the location of supply and the location of clients, taking account of client transportation resources and travel time, distance and cost [9];
- *Accommodation* as the relationship between the manner in which the supply resources are organised to accept clients (including appointment systems, hours of operation, walk-in facilities, telephone services) and the clients’ ability to accommodate these factors [9];
- *Affordability* as the relationship between the prices of services and providers’ insurance or deposit requirements and the client’s income, ability to pay and existing health insurance. Client perception of worth relative to total cost may be a concern, as is clients’ knowledge of prices, total cost and possible credit arrangements [9];
- *Acceptability* as the relationship, between clients’ attitudes about personal and practice characteristics of existing providers including age, sex, location and type of facility or religious affiliation of the provider or facility, as well as provider attitudes about acceptable personal characteristics of clients, including ethnicity and source of payment [9].

In addition to the five accessibility dimensions, data extracted from the included publications consisted of study design, population, location, healthcare service type and main findings. These results were then combined into a single table.

## Results

**Fig. 1:**
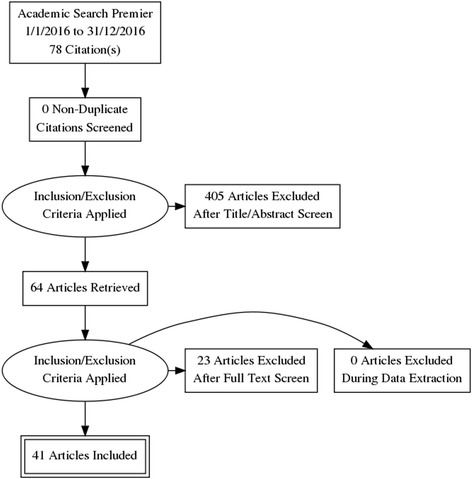
PRISMA flowchart showing study selection for the review of health care accessibility by older Australians

Figure 1 shows the PRISMA flowchart of paper inclusion for the review. Initially, 64 papers were identified in the review. Of these, 23 did not contain any information on the accessibility to health care services in Australia and were discarded. Table 1 provides a summary of the 41 publications included in the study. Eighteen studies were cross-sectional, three were longitudinal, ten were reviews, nine were editorials/commentaries and one was a case study. When looking at specific populations of older Australians included in the studies, six studies looked at migrants and refugees, seven investigated rural and remote populations and one exclusively studied Indigenous populations.

**Table 1:** Summary of included studies

Publication	Study design	Population	Location	Service type	Main finding
Abed et al. (2013) [24]	Systematic review	Older Arab migrants	Australia	Healthcare for older Arab migrants.	We need ‘more culturally competent care’
Abed et al. (2014) [30]	Editorial	Older Arab migrants	Australia	Health services	‘Increase our awareness of cultural attributes, migration experiences, and health perceptions’
Allen et al. (2012) [31]	Self-reported postal survey	Aged 55 years and over	New South Wales	Social support	‘Not being married or de facto relationship, lower education and decreased social support significantly predicted psychological distress’
Anderson et al. (2006) [20]	Opinion piece	Aboriginal Australians	Australia	Health services	‘There is still no needs-based expenditure (in global terms) for Aboriginal health in Australia’
Aoun et al. (2013) [32]	Longitudinal study	Terminally ill people.	Western Australia	Hospice care	‘The ability to die in the place of choice should be looked at as a possible quality measure in end-of-life care’
Banbury et al. (2014) [17]	Systematic literature	Rural and remote communities	Australia	E-health	‘E-health has the potential to increase access to services in rural and remote communities’
Bhar (2016) [33]	Survey	Residential aged care staff	Australia	Psychological services	‘Access to psychologists and psychological services remains poor within Australian residential aged care facilities’
Brand et al. (2011) [34]	Review	People with osteoarthritis of the hip and knee	Australia	Health services needed for patients with osteoarthritis of the hip and knee.	‘Clinicians have evidence-based recommendations for OA management but are poorly supported by service models to deliver these effectively and efficiently’
Cornell (2016) [35]	Opinion piece	Older Australians	Australia	Housing	‘Seeking to optimise outcomes for older people in the context of choice, independence, housing security, participation in community life and wellbeing and assist them to age in place’
Davis (2016) [11]	Systematic review	Older people	Australia	Aged care	‘There is limited high-quality research investigating the effectiveness of interventions at the health and aged care interface of subacute care’
Dellemain et al. (2013) [13]	Opinion piece	Rural people	Australia	Case management	‘There is confusion on the definition of case management, thereby limiting its accessibility to emerging rural services’
Drummond et al. (2011) [14]	Survey	West African refugee women	Perth, Western Australia	Health care services	‘Shame or fear of what family and friends might think, fear of being judged by the treatment provider, fear of hospitalization, and logistical difficulties were significant impediments to accessing health care services’
Evans (2013) [36]	Cross-sectional survey design	Older Australians	South Australia	Respite services	‘The Findings clearly highlight the dual client focus of respite, although there are differences in the nature of the service that is provided to clients’
Feist et al. (2010) [29]	Survey	Aged 55 years and over	Murray Lands, South Australia (rural area)	Technology to link older people to community	‘Attitudes to new technologies varied by age, but positive attitudes were expressed across all ages’
Giles et al. (2009) [10]	Analysis of secondary data	Older Australians	Australia	Aged care	‘Overall the distribution of services available to older persons in uneven across Australia. It will not be adequate to address the increasing needs associated with the ageing of the Australian population’
Greaves et al. (2009) [16]	Longitudinal qualitative, interpretive study using a case study approach with in-depth interviewing.	Socially isolated and unwell older people.	Metropolitan Brisbane.	Community-based aged care.	‘Fear emerged as a common experience embracing aspects of daily life such as depletion of social networks, being dependent on others, loss of mobility and diminishing ability to drive. Inadequate or unreliable public transport resulted in extended waiting times to attend medical appointments’
Harris et al. (2012) [37]	Secondary analysis of a survey	Primary care practitioners	Australia	General practice	‘Improving after-hours access requires a comprehensive approach which includes incentives, improvements to information management and organised systems of care with review if data on clinical outcomes’
Hassett et al. (1999) [27]	Cross-sectional study	Non-English-speaking backgrounds and English-speaking backgrounds	North-west and western metropolitan Melbourne	Acute psychogeriatric	‘Under recognition of disorders such as depression and reluctance to accept necessary inpatient management are two possible factors that should concern mental health service providers for the ethnic elderly’
Hiruy et al. (2014) [38]	Case study	Africans in Australia	Australia	Palliative care	‘The importance of paying sufficient attention to a diverse range of factors including the migration history when providing palliative and hospice care for patients’
Hughes (2007) [39]	In-depth narrative interviews	Older gays and lesbians	Blue Mountains (rural area)	Health and aged care services	‘In addition to direct discrimination, participants reported a more indirect form of discrimination in providers’ assumption of heterosexuality among clients and their failure to provide lesbian or gay-friendly services’
Hughes (2011) [23]	Critical review	Australians	Australia	Aged care, social work	‘There is a need for greater leadership among social work and its representative groups to assert its contribution to aged care, and more broadly to promote the health and wellbeing of all older people’
Hurley et al. (2013) [15]	Telephone interviews with formal service providers, and interviews and focus groups with Greek elders.	Greek elders	Adelaide, South Australia	Community-based services	‘Formal service providers need to ensure that services are promoted and delivered to take account of the important role of family in informal support while also addressing the access, challenges posed by language and literacy’
Jeon et al. (2012) [19]	Semi-structured interviews via postal survey	Members of National Seniors Australia over 50 years of age	Australia	Chronic care services	‘… findings highlight the degree to which people whose resources are constrained are prepared to go to maintain access to private hospital care’
Jiwa et al. (2013) [40]	Opinion piece	Australians	Australia	General practitioner	‘Australians have greater access to the internet than ever before. With a little more investment this technology could facilitate online video-consultations with general practitioners’
Joo et al. (2013) [41]	Integrative review of literature	Whole population	International including Australia	Community-based settings	‘Community based case management significantly reduced hospital access outcomes, especially readmissions and increased cost effectiveness, patient clinical outcomes and patient satisfaction’
Lau et al. (2012) [25]	Opinion piece	Older people	Australia	Palliative care	‘Ethnic minorities, older people and patients with non-cancer diseases are found to be at a greatest risk for underutilisation of palliative care. Barriers to access palliative care by these groups in the community are complex and often overlapping’
Lowe (2011) [42]	Editorial	Women	Australia	Cardiovascular health services	‘Perhaps in a health system that fails to deliver cardiovascular prevention well for anyone, women, who are not only more likely to be older and frailer, but also more likely to be economically and educationally disadvantaged than men, are less likely to get preventative care’
Lowthian et al. (2012) [18]	Exploratory descriptive study of structured interviews	Lower urgency community-dwelling patients aged 70+ years	Metropolitan Melbourne, Australia	Public hospital emergency department	‘Emergency departments should be redesigned and/or integrated community-based models of care developed to meet the specific needs of this age group who have growing demand for acute care’
Moorin et al. (2012) [43]	Cross-sectional study using administrative records	Australians (including those aged ≥ 65 years)	Australia	Cancer service	‘Cancer services are not provided uniformly (horizontal equity) across strata of socio-economic status’
Muir-Cochrane et al. (2014) [12]	Semi-structured interviews	Older people (aged 65 and over)	Rural Australia	Mental health	‘This study offers new insight into the difficulties that arise from the separation of physical and mental health systems for older people with multiple needs, and the impact of living in a rural region on unmet mental health care needs of older people’
Rosenwax et al. (2015) [44]	Retrospective population-based cohort study	People in their last year of life	Western Australia	Emergency department	‘Decedents with dementia who were not receiving community-based palliative care attended hospital emergency departments more frequently than people receiving community-based palliative care’
Russi (2014) [45]	Opinion piece	People living with disability	Australia	Disability	‘Many inequities faced by people living with disability could be reduced by the introduction of the National Disability Incentive Scheme through the equitable access to services and supports and removal of financial barriers’
Schofield (2008) [46]	Opinion piece	Australians	Australia	Health services	‘Research and policy tools for analysing, understanding and fixing the problem are crucial’
Tabrizi et al. (2008) [21]	Cross-sectional study	Type II diabetics	Queensland, Australia	Clinical care	‘Service quality including choice of care provider, accessibility, prevention, continuity, timeliness and safety were identified to be of inadequate quality’
Tang et al. (2011) [26]	Narrative review	Older Australians	Australia	Respite	‘Respite care needs to move away from a custodial model to a more psychological model of care, and that more natural and flexible models (e.g. host family respite), integrated with increased post-respite support and psychosocial education’
Tilse (2002) [47]	Semi-structured interviews	Management, families and residents	Rural Australia	Aged care facilities	‘The complexity of current financial arrangements, access to appropriate financial advice at the time of entry, and the potential for an informal two-tier system in relation to the allocation of amenities are identified as developing policy issues’
Walker (2012) [22]	Literature search	People with multiple conditions	Australia	Chronic conditions health services	‘Services and policies require specific reforms to better meet the needs of people with multiple conditions’
Warburton et al. (2015) [48]	Multistage mixed-methods	Practitioners	Rural Australia	Specialist service organisations	‘The challenges of rural assessment, are both demand-driven and supply issues’
Ward et al. (2011) [49]	Mixed methodology including quantitative and qualitative methods	35 to 75 years of age	South Australia	Bowel screening	‘The main system-related barriers were the lack of awareness of colorectal cancer or screening. The problems with language due to most of the information being in English and the lack of recommendation by a doctor’
Wark et al. (2015) [50]	Semi-structured interviews	Older adults and carers	Rural Australia	Disability services	‘An understanding of the needs of older adults with learning disability resident in rural areas is important to ensure that both aged-care and disability support structures are built on individuals’ needs’
Wark et al. (2015) [28]	Delphi conducted over three rounds	Disability workers who support people with learning disability	Rural New South Wales, Australia	Non-government disability services	‘A thematic analysis indicated three main themes of access to services; time constraints and funding’

The results of the analysis of papers against Penchansky and Thomas’ five dimensions of accessibility [9] are shown in Table 2. Each of the dimensions of accessibility were represented in the review with 22 papers identifying issues of *acceptability*, 17 papers *availability*, 16 papers *accessibilit*y, 12 papers *affordability* and 6 papers *accommodation*. Of note is that 22 papers out of the 41 papers reviewed described more than one dimension of accessibility.

**Table 2:** Healthcare accessibility of included studies according to Penchansky and Thomas’ five dimensions of accessibility

Publication	Availability	Accessibility	Accommodation	Affordability	Acceptability
Abed et al. (2013) [24]					X
Abed et al. (2014) [30]					X
Allen et al. (2012) [31]		X			
Anderson et al. (2006) [20]	X			X	X
Aoun et al. (2013) [32]					X
Banbury et al. (2014) [17]	X			X	
Bhar (2016) [33]	X		X	X	
Brand et al. (2011) [34]					X
Cornell (2016) [35]			X		X
Davis (2016) [11]	X				
Dellemain et al. (2013) [13]	X	X	X		
Drummond et al. (2011) [14]		X		X	X
Evans (2013) [36]		X			X
Feist et al. (2010) [29]	X	X			
Giles et al. (2009) [10]	X	X			
Greaves et al. (2009) [16]		X			
Harris et al. (2012) [37]	X		X		
Hassett et al. (1999) [27]					X
Hiruy et al. (2014) [38]					X
Hughes (2007) [39]					X
Hughes (2011) [23]	X				X
Hurley et al. (2013) [15]	X	X		X	X
Jeon et al. (2012) [19]				X	
Jiwa et al. (2013) [40]	X			X	
Joo et al. (2013) [41]				X	
Lau et al. (2012) [25]					X
Lowe (2011) [42]		X			
Lowthian et al. (2012) [18]			X		
Moorin et al. (2012) [43]	X	X		X	
Muir-Cochrane et al. (2014) [12]	X	X	X		X
Rosenwax et al. (2015) [44]		X			
Russi (2014) [45]	X			X	
Schofield (2008) [46]					X
Tabrizi et al. (2008) [21]	X		X		
Tang et al. (2011) [26]	X	X		X	X
Tilse (2002) [47]				X	X
Walker (2012) [22]					X
Warburton et al. (2015) [48]		X			
Ward et al. (2011) [49]					X
Wark et al. (2015) [50]		X			X
Wark et al. (2015) [28]	X	X			

## Discussion

This review has highlighted a number of factors affecting older Australians’ access to health services. Penchansky and Thomas’ dimensions of accessibility have provided a good working framework to organise the issues [9].

### Availability

The availability of health services was often reported as an uneven distribution of health services not only in terms of spatial distribution but also with regards to the type of services offered for continuity of care [10]. For instance, there was an undersupply of many health services in both urban and rural areas, particularly the latter. Many older Australians were found to need to travel to services or wait for health professionals to visit. Of concern, an undersupply of acute and subacute hospital services was commonly reported [11] and, to a lesser extent, access to aged care services.

Another barrier to older Australians accessing health services was the lack of knowledge of what services were available and where to access them [12]. This barrier was not only experienced by patients and their families but also by health professionals and other service providers [12]. As well as knowing what services are available, access to health services was found to be initiated through a referral from a general practitioner (GP) [12], with many services not taking on patients without one, therefore creating a barrier for collaborative care.

### Accessibility

This review has also identified a number of geographic accessibility issues facing older Australians accessing health services, particularly due to low workforce numbers, a lack of services and poor infrastructure [13]. Geographic accessibility to health services is a multifaceted issue for older Australians with transport to cover the geographic distance to health services posing multiple barriers for older people [14]. Older people with no transport are reliant on either family members or other forms of transport to access services [15]. The unreliable and/or non-existent public transport in many areas limits access to services [12]. Those that do drive prefer to drive at certain times and shorter distances, with preferences not to drive in heavy traffic or late in the day limiting their availability to accept appointments offered outside of their referred times [16].

As well as the direct issues associated with transport for older Australians accessing health services, there are boarder travel issues for many. The financial burden of travel can be significant for those travelling long distances especially when accommodation is required [17]. Fear of travel can be a concern for older Australians which includes fear of leaving home and family support networks [17].

E-health was seen within the literature as an alternative to traveling long distances to access health services. E-health was discussed as a way of enabling older people to access health care within their local area and therefore reducing the travel costs and the physical and practical inconveniences associated with having to travel to services [17].

### Accommodation

This review also found that a lack of services, long waiting times and difficulties in getting an appointment with GPs and specialist health professionals were significant barriers for older people [18]. This is particularly an issue in rural areas where there is a lack of workforce due to government policy and difficulties in attracting families to rural areas limiting services [13]. Many health services are at capacity and after-hours appointments are limited or not available [12]. This is an issue when GPs act as a gateway to other health services.

### Affordability

Penchansky and Thomas define affordability as the relationship between the prices of services and providers’ insurance or deposit requirements and the client’s income, ability to pay and existing health insurance [9]. Client perception of worth relative to total cost may be a concern, as is clients’ knowledge of prices, total cost and possible credit arrangements.

Older Australians often bare a larger financial burden than younger Australians due to the management of multiple chronic conditions and being reliant on a reduced fixed income [19]. However, the Medicare System in Australia provides free access to public hospital services and pays a scheduled amount for private medical services and ancillary health services [19]. The Pharmaceutical Benefits Schedule (PBS) subsidises the cost of pharmaceuticals to all Australians with the highest subsidies going to those on low incomes [20]. Both of these schemes aid in the affordability of health care for older Australians; however, many health professionals charge gap payments which make services less affordable. This review found that although Australians are able to claim five annual allied health service visits through Medicare when managed by their GP, many older Australians with ongoing health issues, five annual consultations were insufficient to enable effective management and active participation in self-care, productivity and leisure tasks and roles [21]. The current disability support system in Australia was described in the literature as underfunded, unfair, fragmented and inefficient [21]. E-health was reported in the literature as being a cost-effective method to deliver health services and remote monitoring in rural and remote areas [17].

### Acceptability

Penchansky and Thomas define acceptability as the relationship, between clients’ attitudes about personal and practice characteristics of existing providers including age, sex, location and type of facility or religious affiliation of the provider or facility, as well as provider attitudes about acceptable personal characteristics of clients, including ethnicity and source of payment [9].

The literature review has highlighted that patient perceptions on the separation of physical and mental health service provision is an issue with the lack of collaboration between health services resulting in people with complex interconnecting physical and mental health problems having difficulties accessing services for an appropriate continuum of care [12]. There is also a need for services to be not only clinical but also broader in nature to include social support, informal and in-home services [22].

Health care for older people from ethnic minorities is seen as challenging because they tend to have a higher prevalence of chronic and disabling disease, and there is limited knowledge of their health care needs. The literature review has identified that older patients from culturally and linguistically diverse backgrounds are not a homogenous group and that this in itself creates many challenges for delivering appropriate models of health care through health services which fit their specific cultural needs [23]. Health care for older people from ethnic minorities can be challenging due to knowledge, attitudes, beliefs and health-seeking behaviours [24]. Central to older people accessing health care is the role of the family, duty and respect for parents and older people, religion, feelings of shame when receiving care from a non-family member, gender sensitivity and the difficulties of combining western health practices with their traditional health practices [14, 24–26].

Limited proficiency in English is frequently cited as the most problematic factor for the ethnic elderly in accessing mental health and other aged care services [27]. This is not only a problem for recently arrived elderly migrants, but also for many who came to Australia in the post-World War II migration boom [27]. While many older patients from ethnic backgrounds prefer to use family members as mediators and interpreters when dealing with health services, this often conflicts with organisational patient privacy policies [28].

The findings from this review have important implications for both service providers and health care policy makers. Australia is in the midst of a major health and aged care reform, and knowledge of older adults’ accessibility to healthcare services can better position health, aged and social care services to tackle future challenges expected from population ageing. For instance, identifying specific accessibility issues to care services and why these accessibility issues are occurring can inform health resource allocation decisions made by state and federal health departments; identifying locations for future health services, new mobile services, telehealth and other improvable components such as opening hours and costs of services are important factors that should be considered.

Results from this review are also relevant to older adults themselves. Now and into the future, the care needs of older adults need to be tailored to the individual beliefs, priorities and preferences of older adults. Consideration of these factors will enhance health service accessibility and, in turn, improve both service integration and outcomes of care.

## Conclusion

The literature review has identified that currently older Australians face a number of accessibility issues when accessing health care services within Australia. With the increase in the number of older Australians and their preferred choice to age in place, health service planning will need to become more patient-centred. The literature review has highlighted the contribution of factors to health service accessibility that varies according to an older person’s geographical local, their accessibility to transport and their level of multi-morbidity and cultural background. Understanding the three important dimensions to ageing-in-place, the home the older person resides in, the community the person interacts within and the services and support that are available to them [29], will need to shape current and future health service models.

### Limitations

This review identified issues facing older Australians accessing health services. Although the database search was comprehensive, it was limited to English-language papers published in scientific journals located by Academic Premier. Grey literature was also not searched, which may have provided an additional insight into issues affecting the health service accessibility of older adults. A further limitation of this review is that the majority of included publications were either cross-sectional or opinion pieces. Whilst these were informative, it is recommended that additional longitudinal study of healthcare accessibility for older Australians be performed as a means to further elucidate the issues underlying healthcare accessibility. Moreover, although there were several studies of older migrants/refugees identified in our review, there was a distinct lack of studies looking at the healthcare accessibility of Indigenous older Australians. Thus, given the large Indigenous gap in both health and life expectancy in Australia [1], it is recommended that further studies investigate healthcare accessibility issues of older Indigenous Australians.
